# Prevalence, awareness, treatment and control of hypertension and sodium intake in Jiangsu Province, China: a baseline study in 2014

**DOI:** 10.1186/s12889-016-2712-y

**Published:** 2016-01-21

**Authors:** Zhang Yongqing, Wu Ming, Su Jian, Luo Pengfei, Pan Xiaoqun, Dong Meihua, Lou Peian, Dong Jianmei, Zhou Guoyu, Yang Jie, Lin Ping, Xu Yan

**Affiliations:** 1 Jiangsu provincial Center for Disease Control and Prevention, 210009 Nanjing, China; 2 Wuxi Center for Disease Control and Prevention, 214000 Wuxi, China; 3 Xuzhou Center for Disease Control and Prevention, 221000 Xuzhou, China; 4 Lianyungang Center for Disease Control and Prevention, 222000 Lianyungang, China; 5 Yangzhou Center for Disease Control and Prevention, 225000 Yangzhou, China

## Abstract

**Background:**

The prevalence of hypertension in Chinese Mainland has increased rapidly in the recent decades. This study aimed to determine the prevalence of hypertension and sodium intake in an economically booming area in China.

**Methods:**

9600 adults aged from 18 to 69 years old in Jiangsu Province of China were recruited using a complex, multistage sampling method. Blood pressure was measured and a face-to-face interview was conducted among all participants. 24 hours (24-h) urine sample was collected from each participant and then measured for sodium and potassium. Hypertension was determined by blood pressure and use of anti-hypertension medications. All of the analyses were weighted according to the population distribution in the province.

**Results:**

Overall, the weighted means of systolic blood pressure (SBP) and diastolic blood pressure (DBP) were 128.8 mm Hg (95 % confidence interval, CI, 128.3–129.3) and 82.2 mm Hg (95 % CI, 81.4–83.1). The weighted hypertension prevalence of Jiangsu residents was 33.0 % (95 % CI, 29.4–36.7 %). Among those with hypertension, 31.4 % (95 % CI, 24.6–38.1) were aware of their blood pressure condition. In total, 88.4 % (95 % CI, 83.5–93.3) of those with known hypertension took anti-hypertension medications. Only 23.7 % (95 % CI, 13.3–34.2) of those under anti-hypertension medications had their blood pressure controlled. The mean of 24-h urinary sodium excretion was 188.2 mmol (standard deviation, SD, 69.5), representing that the mean intake of salt was 11.0 g (SD, 4.1) through conversion.

**Conclusion:**

Hypertension and excessive sodium intake in adults are prevalent in Jiangsu Province, China. These observations suggest that a public health approach is necessary to prevent hypertension and manage hypertensive patients.

**Electronic supplementary material:**

The online version of this article (doi:10.1186/s12889-016-2712-y) contains supplementary material, which is available to authorized users.

## Background

Hypertension is a major health problem worldwide. More than 60 % of stroke cases and 40 % of coronary heart disease events were attributed to hypertension worldwide [1]. The prevalence of hypertension in general population was approximately 25 %, and this number will increase markedly by 60 % between 2000 and 2025 [2]. Pre-hypertension, a physiological condition that blood pressure is not as high enough as that defined as hypertension clinically, is also revealed as one important cause of global mortality [3].

Reduced salt intake 50 mmol per day will decrease systolic blood pressure 4 mmHg and diastolic blood pressure 2.5 mmHg [4]. Excess dietary sodium is closely related to the increase of blood pressure and clinically measured hypertension. Furthermore, because excess sodium can increase urinary calcium loss, it may also cause many other adverse health outcomes such as ventricular fibrosis, renal damage, gastric cancer, and osteoporosis [5].

Jiangsu province lies in the eastern region of China with a population of 73 million. In the province, cancer and cardiovascular disease are the two major death-related causes around the residents [6]. According to the monitoring data from “The Chronic Disease And Its Risk Factors In Jiangsu Province, 2010”, the prevalence of hypertension in residents aged 18 and older in Jiangsu province has reached 38.6 %. In addition, the average level of daily salt consumption (11.2 g) was almost 2 times of the World Health Organization guideline (6 g) [7].

Since 1970s, many countries launched campaigns to reduce salt intake. Salt reduction is considered as a prior measure to decrease the occurrence of cardiovascular disease and the medical expense [8, 9]. The health benefits of salt reduction action have been well documented. Measures supported by policy, such as creating supporting environment, large-scale public education, adjusting the formulation and implementation of “salty food label”, can gradually reduce the sodium intake of population [10]. These experiences show that the health promotion strategies as comprehensive measures for salt intake reduction can prevent high blood pressure and related diseases consequently.

The awareness, treatment and control of hypertension in treated patients increased significantly from 1997 to 2009. However, these observed rates were all less than 30 % [11]; so the current measures for preventing and treating hypertension still required to be enhanced. As a result, Jiangsu provincial government started a salt reduction action on July 29, 2013, this project was led by the provincial government and was aiming to reduce the risk of hypertension and related diseases; and its baseline survey was carried out to understand the level of population sodium intake and hypertension prevalence around the whole province in 2013. These findings can improve a targeting intervention to achieve the health goal of government: the awareness of salt knowledge reaching more than 80 % and the provincial average level of salt intake decreasing by more than 28 % at the end of 2015.

## Methods

### Sample size and sampling frame

The baseline survey of National Salt Reduction Action was conducted between December 2013 and May 2014. Respondents were restricted in persons who aged 18 to 69 years old and lived in the selected areas for 6 to 12 months before the investigation. The sample size used for estimating hypertension prevalence of the provincial population was calculated as 9600, its calculation formula was $\mathrm{N}=\frac{u_{\alpha}^2p\left(1\mathit{\hbox{-}}p\right)}{\delta^2}$, and all parameters in this formula were determined by the project plan; The required sample size was 2400 for salt intake evaluation, the calculation formula was $\mathrm{N}={\left(\frac{u_{\alpha}\sigma }{\delta}\right)}^2$ and parameters in this formula also were determined by the project plan. Participants without disability and mental disorders were eligible to enter into the survey.

**Fig. 1:**
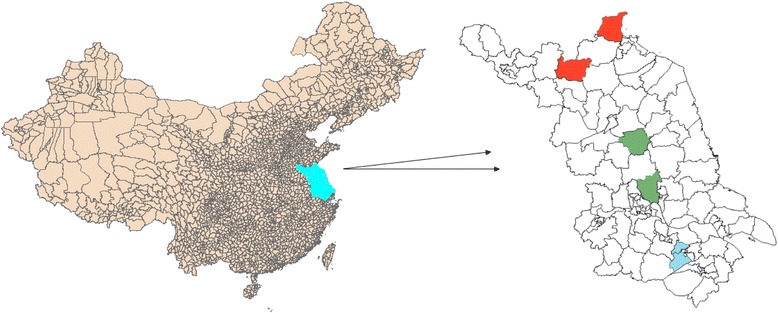
Location of the sampled countries/districts in Jiangsu Province, China, Baseline Results From the National Salt Reduction Action in Jiangsu Province, 2013. [13 prefecture-level cities in Jiangsu province are traditionally divided as north North Jiangsu (Xuzhou, Lianyungang, Suqian, Yancheng and Huaian), middle Middle Jiangsu (Yangzhou, Taizhou and Nantong) and south South Jiangsu (Nanjing, Zhenjiang, Changzhou, Wuxi and Suzhou) with geographic distribution and economic status]

**Fig. 2:**
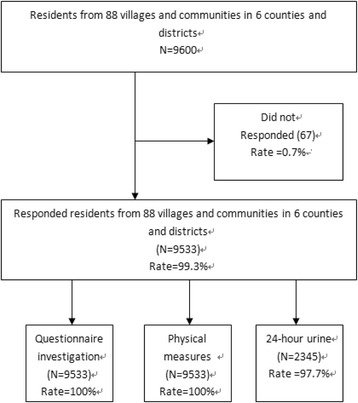
Participants in the baseline survey of the National Salt Reduction Action in Jiangsu Province, China, in 2013

We used complex four-stage cluster sampling method to recruit participants.

(1). Stage 1, 6 counties/districts were selected from 100 counties/districts following geographic distribution and residence status (Binhu and Huishan district of Wuxi city represent South Jiangsu, Jiangdu district and Baoying county of Yangzhou city represent Middle Jiangsu, Xinyi county in Xuzhou city and Ganyu county in Lianyungang city represent North Jiangsu). The geographic location of 6 selected counties/districts is shown in Fig. 1.

(2). Stage 2, 4 towns (streets) were selected from each county/district by using proportional probability sampling.

(3). Stage 3, we also used proportional probability sampling to select 3 villages (neighborhood) randomly from selected towns (streets) in Middle and South Jiangsu, and 5 villages (neighborhoods) from selected towns (street) in North Jiangsu.

(4). Stage 4, we randomly selected 100 adults from the local resident list in each selected village and neighborhood community in Middle and South Jiangsu, and 120 adults in North Jiangsu. A total of 9600 participants aged 18 to 69 years old were selected from 9600 households among 6 communities and villages. In each selected village, 25 (from the middle and south) and 50 (from the north) of the participants were selected for urine test. Overall, the urine samples from 2400 adult participants were collected for further laboratory tests. The details of sampling procedure from stage 1 to stage 4 are shown in Fig. 2.

### Measurements

This baseline survey includes questionnaire investigation, physical measurement and laboratory tests. The questionnaire investigation and physical measurement were recommended for all selected participants. The amounts of chemicals in the urine (a 24-h urine sample collection) were subsequently determined by laboratory tests.

#### Questionnaire investigation

All of the families were invited to finish the family questionnaires containing family economic status and dietary information. A personal questionnaire (details in additional file 1) including individual social demography, hypertension related diseases conditions (ie. hypertension, diabetes, stroke and coronary heart disease), related lifestyle (smoking, alcohol use, diet and physical activity), related knowledge about salt intake and hypertension (health outcomes of sodium intake and hypertension, perceptions of salt consumption attitude and behaviors toward reducing salt intake), was answered by selected subjects.

#### Physical measurements

All respondents were invited to carry on the physical measurements including blood pressure, height, weight, waist circumference, hip circumference. These indexes were measured by well-trained health staffs using national standard protocols [12]. For instance, electronic sphygmomanometer (HEM-7071, Omron Corporation, Kyoto, Japan) is required for the blood pressure measurement, and the participants must keep in the sitting position during the test. One valid test should repeat every 5 min for 3 times by a single surrounding.

Body mass index (BMI) = weight (kg) / height ^2^ (m), the definition of BMI categories is including: BMI less than 18.5 is classified as lower weight, BMI ranging from 18.5 to 24.99 is classified as normal, BMI ranging from 25 to 28 is classified as overweight and BMI more than 28 is classified as obesity.

#### Laboratory tests

The 24-h urine specimens of respondents were collected in standard urine collection containers. After the first interview when the participants were given collection instructions, the beginning time and ending time of urine collection for each participant were recorded and the completeness of 24-h urine collection was evaluated [1]. Then, the containers of all respondents were taken back and then transported on ice to the laboratory (ADICON Clinical Laboratory Inc., Jinan, Shandong, China), where the experiments were carried out according to the testing methods. Urinary sodium, urinary potassium and urinary creatinine were determined by the designated inspection agencies [13].

Urinary sodium and potassium were measured using ion selecting electrode method in accordance with the examination protocols. Creatinine, as a quality control index, was measured using the picric acid method. The completeness of 24-h urine collection was assessed based on creatinine excretion. The urine sample with creatinine reaching 1.91–18.27 mmol in man or 1.36–14.28 mmol in woman was considered an acceptable collection [14]. Based on assumption that all ingested sodium was in the form of sodium chloride, quantity of 24-h urinary sodium excretion was used estimating daily salt intake [1].

Ethical approval of this study was obtained from the “Ethical Committee of Chinese National Center of Disease Control and Prevention”. And all respondents in this investigation had received and signed a papery informed consent form from Ethical Committee of Chinese National Center of Disease Control and Prevention.

#### Hypertension definition

The participant was classified as hypertension if systolic blood pressure (SBP) was greater than or equal to 140 mm Hg, or diastolic blood pressure (DBP) was greater than or equal to 90 mm Hg, or he/she underwent anti-hypertension medication in the previous 2 weeks. Among the hypertensive participants, self-report of any previous clinical diagnosis of hypertension was defined as awareness of hypertension. Self- reported anti-hypertension medication usage was defined as treatment of hypertension among the participants with hypertension awareness. Finally, mean SBP of less than 140 mm Hg or DBP of less than 90 mm Hg was defined as control of hypertension among those treated participants [15].

### Data analysis

Sample weights were determined by the design weight and post-stratification weight. The design weight was calculated by combination of cluster design, strata and individual. And the calculation of post-stratification weight depended on general population distribution of Jiangsu Province in 2014 [16].

All of the data in the survey were collected and inputted by using EpiData Entry 3.1 (The EpiData Association, Odense, Denmark). Statistical analysis were performed with SPSS (version 19.0, SPSS Inc., Chicago, Illinois, USA) and SAS software 9.3 (SAS Inc., Cary, North Carolina, USA). When *P* value was less than 0.05 (Two sided *P* < 0.05), the difference was considered statistically significant.

Results were expressed as median and 95 % confidence interval (95 % CI) or mean ± SD. Variances of mean and proportion were estimated and 95 % confidence intervals (CIs) were calculated. Rao-Scott *χ* ^2^ test was used for comparisons of rates between different characteristics such as household registers, sexes and age groups, while Student’s *t*-test and analysis of variance (ANOVA) were used for comparisons of means between different characteristics.

## Results

### Characteristics of respondents

Of the 9600 individuals invited to this study, 9533 individuals (4650 men, 4883 women) responded and participated in our survey. The response rate is 99.3 %. A total of 2202 individuals completed the 24-h urine collection, yielding a response rate of 91.8 %.

The mean age of the samples was 41.55 (SD, 13.797) years old. Of the participants, 2620 (27.9 %) participants were current smokers. The prevalence of overweight and obesity was 36.6 and 14.6 %, respectively. Based on the body mass index (BMI), 264 (3.1 %) participants were classified as low weight (BMI < 18.5), 4344 (45.7 %) were normal (18.5≦BMI < 25), 3496 (36.6 %) were overweight (25≦BMI < 28), and 1410 (14.6 %) were obese (BMI≧28). All details of respondents’ characteristics are shown in Table 1.

**Table 1:** Characteristics of study participants in different sex of Jiangsu population

Characteristic		Total			Urban			Rural			Male			Female		
		N	%	95 % CI	N	%	95 % CI	N	%	95 % CI	N	%	95 % CI	N	%	95 % CI
Age (years)	18–34	3281	35.7	30.1–41.4	1206	42.2	35.7–48.7	2075	31.4	23.0–39.8	1592	35.7	29.3–42.0	1689	35.8	30.7–40.8
	35–49	3394	35.2	32.0–38.4	1312	34	30.6–37.6	2082	36	31.3–40.7	1632	34.9	30.8–39.0	1762	35.5	33.1–37.9
	50–69	2858	29.1	25.6–32.6	1060	23.8	18.9–28.9	1798	32.6	27.7–37.5	1426	29.4	25.8–33.1	1432	28.7	25.3–32.1
Ethnicity	Han	9508	99.7	99.6–99.9	3570	99.8	99.6–100	5938	99.7	99.6–99.8	4646	99.9	99.8–100	4862	99.6	99.4–99.8
	Other	25	0.3	0.1–0.4	8	0.2	0.0–0.4	17	0.3	0.2–0.4	4	0.1	0–0.2	21	0.4	0.2–0.6
Education (years)	0	1434	14.7	12.5–17.0	118	3.1	0–7.9	1316	22.5	20.1–24.8	374	7.8	5.7–9.9	1060	21.7	19.7–23.7
	1–5	1618	16	14.2–18.0	454	11.2	5.3–17.0	1164	19.4	18.1–20.7	695	14.4	12.8–16.1	923	17.7	15.2–20.3
	6–8	3625	36.8	33.4–40.3	1303	35	27.8–42.1	2322	38.1	34.7–41.5	1916	39.9	34.9–44.9	1709	33.8	30.8–36.6
	9–11	1716	18.7	16.6–20.9	827	23.9	20.4–27.5	889	15.3	12.7–17.9	1034	22.8	20.5–25.2	682	14.6	12.6–16.7
	>12	1134	13.6	9.1–18.1	876	26.8	14.8–38.9	258	4.8	0.3–9.3	527	15	10.0–20.0	507	12.2	7.6–16.8
Smoking status	Current	2620	27.9	25.3–30.5	958	26.4	20.0–32.7	1662	28.9	27.4–30.4	2540	53.9	49.2–58.6	80	1.8	0.7–2.9
	Former	251	2.6	1.6–3.5	85	1.9	0–3.9	166	3	2.5–3.6	242	5	3.1–6.8	9	0.2	0–0.3
	Never	6653	69.5	66.6–72.4	2535	71.7	65.1–78.4	418	68.1	66.0–70.1	1868	41.1	36.0–46.3	4785	98	97.1–99.0
BMI	Low weight	264	3.1	2.3–3.8	148	4.6	2.5–6.7	116	2	1.3–2.8	103	2.4	1.6–3.2	161	3.7	2.8–4.7
	Normal	4344	45.7	41.3–50.1	1751	49.2	41.4–57.1	2593	43.4	37.2–49.5	2000	42.3	36.1–48.8	2344	49	44.2–53.8
	Overweight	3496	36.6	33.2–40.1	1244	34.1	27.8–40.4	2252	38.3	33.5–43.1	1825	39.5	34.9–44.2	1671	33.7	30.7–36.7
	Obese	1410	14.6	12.2–17.0	432	12	8.0–16.0	978	16.3	12.9–19.7	715	15.6	13.3–17.9	695	13.6	9.3–17.8

### Blood pressure and awareness, treatment and control of hypertension

Overall, the means of SBP and DBP were 128.8 mmHg (95 % CI, 128.3–129.3) and 82.2 (95 % CI, 81.4–83.1) mmHg, respectively. Men had higher SBP (132.2 mmHg (95 % CI, 131.1–133.3) VS 125.4 (95 % CI, 124.2–126.7) mmHg) and DBP (84.3 mmHg (95 % CI, 82.9–85.7) VS 80.1 mmHg (95 % CI, 79.4–80.9)) than women. Urban residents had lower SBP (124.6 mmHg (95%CI, 122.2–127.0) VS 131.6 mmHg (95 % CI, 131.1–132.1)) and DBP (81.3 mmHg (95 % CI, 79.5–83.1) VS 82.8 mmHg (95 % CI, 82.0–83.7)) than rural residents.

The hypertension prevalence of Jiangsu residents was 33.0 % (95 % CI, 29.4–36.7 %). Among those with hypertension, 31.4 % (95 % CI, 24.6–38.1 %) were aware of their blood pressure condition, and 88.4 % of residents with awareness had self-report of taking anti-hypertension medications now; Finally, 23.7 % (95 % CI, 13.3–34.2 %) of residents undergoing anti-hypertension medications had their blood pressure controlled.

The prevalence of hypertension was higher in men than in women (39.0 % (95 % CI, 31.9–46.1 %) VS 27.0 % (95 % CI, 26.1–28.0 %)). There was a significant urban/rural difference in the prevalence of hypertension: 36.2 % (95 % CI, 30.7–41.7 %) for rural residents and 28.2 % (95 % CI, 21.4–35.1 %) for urban residents.

Among those with hypertension, there were significant gender and urban/rural differences in awareness and treatment of hypertension (*P* < 0.05). Men had lower awareness and treatment of hypertension than women, while the urban residents had higher awareness of hypertension than the rural residents (Table 2). The prevalence of awareness and treatment of hypertension was 28.6 % (95 % CI, 19.8–37.5 %) and 86.7 % (95 % CI, 82.2–91.1 %) in men as compared with 35.3 % (95 % CI, 31.0–39.6 %) and 90.4 % (95 % CI, 84.3–96.5 %) in women.

**Table 2:** Means of SBP, DBP and prevalence, awareness, treatment and control of hypertension in Jiangsu Province

Measure	Total		Sex					Urban/Rural				
			Male		Female		P	Urban		Rural		P
	Mean	95 % CI	Mean	95 % CI	Mean	95 % CI		Mean	95 % CI	Mean	95 % CI	
SBP	128.8	128.3–129.3	132.2	131.1–133.3	125.4	124.2–126.7	*P* < 0.05	124.6	122.2–127.0	131.6	131.1–132.1	*P* < 0.05
DBP	82.2	81.4–83.1	84.3	82.9–85.7	80.1	79.4–80.9	*P* < 0.05	81.3	79.5–83.1	82.8	82.0–83.7	*P* < 0.05
Prevalence of hypertension	33.0	29.4–36.7	39.0	31.9–46.1	27.0	26.1–28.0	*P* < 0.01	28.2	21.4–35.1	36.2	30.7–41.7	*P* = 0.03
Awareness of hypertension	31.4	24.6–38.1	28.6	19.8–37.5	35.3	31.0–39.6	*P* < 0.01	41.7	28.1–55.3	26.0	16.7–35.3	*P* = 0.02
Treatment of hypertension	88.4	83.5–93.3	86.7	82.2–91.1	90.4	84.3–96.5	*P*=0.03	90.9	84.3–97.5	86.3	79.4–93.1	*P* = 0.19
Control of hypertension	23.7	13.3–34.2	24.7	14.7–34.8	22.6	11.2–34.0	*P*=0.24	29.3	20.3–38.3	18.8	3.0–34.6	*P* = 0.17

Among the residents who underwent anti-hypertension medications presently, difference in the control of hypertension was not observed between the male and the female (*P* = 0.24), and also not found between the rural and the urban areas (*P* = 0.17). There were no significant gender and urban/rural differences in the control of hypertension. All details of respondents’ blood pressure and awareness, treatment and control of hypertension are shown in Table 2.

### Urinary sodium and potassium

As shown in Table 3, the mean 24-h urinary sodium and potassium excretion were 188.2 mmol (SD, 69.45) and 28.0 mmol (SD, 9.50), respectively. The mean of salt intake converted from urinary sodium was 11.0 g (SD, 4.06). Differences were found in 24-h urinary sodium excretion between different populations. For instance, rural residents excreted more sodium than urban residents (*P* < 0.01); the males excreted more sodium than the females (*P* < 0.01). Among the samples, only 8.6 % (95 % CI, 7.3–9.9 %) had urinary sodium <102.46 mmol (equivalent to salt intake of 6 g/d). All details of respondents’ urinary sodium and potassium are shown in Table 3.

**Table 3:** Urinary sodium and potassium excretion in different populations

24 h Urinary measure		N	Sodium (Mmol/24 h)		Potassium (Mmol/24 h)		24 h Na/K		Converted Salt(g)	
			Mean ± SD	P	Mean ± SD	P	Mean ± SD	P	Mean ± SD	P
Sex	Male	1069	196.36 ± 72.29	*P* < 0.01	28.37 ± 10.07	*P* = 0.07	7.14 ± 2.00	*P* < 0.01	11.49 ± 4.23	*P* < 0.01
	Female	1133	180.47 ± 65.77		27.64 ± 8.92		6.69 ± 1.96		10.56 ± 3.85	
Residence	Urban	823	205.23 ± 71.35	*P* < 0.01	29.97 ± 10.18	*P* < 0.01	7.09 ± 2.07	*P* < 0.01	12.01 ± 4.17	*P* < 0.01
	Rural	1379	178.02 ± 66.26		26.81 ± 8.86		6.80 ± 6.80		10.41 ± 3.88	
Age	18–34	625	189.55 ± 69.94	*P* = 0.19	26.47 ± 8.76	*P* < 0.01	7.30 ± 1.98	*P* < 0.01	11.09 ± 4.09	*P* = 0.19
	35–49	731	190.91 ± 71.66		28.34 ± 9.96		6.92 ± 1.93		11.17 ± 4.19	
	50–69	846	184.82 ± 67.05		28.81 ± 9.49		6.59 ± 2.00		10.81 ± 3.92	
Total		2202	188.19 ± 69.45		27.99 ± 9.50		6.91 ± 1.99		11.00 ± 4.06	

## Discussion

Our study has found that the hypertension prevalence of residents in Jiangsu province was 33.0 % in 2014, as a result, one hypertensive patient was potentially detected from three residents aged from 18 to 69 years old in Jiangsu province. In former studies, the prevalence of hypertension in Chinese population increased significantly from 1991 to 2009 [11], while another surveillance data from Chronic Disease and Its Risk factors Surveillance in China also confirmed this characteristic in Jiangsu province that the standardized rates of hypertension prevalence in residents aged more than 15 years old in 2002, 2007 and 2010 was 25.1, 33.8 and 38.6 %, respectively [17]. In brief, these surveillance data all indicate a tendency of rising hypertension burden in China and Jiangsu province in recent years; Although comparing these hypertension prevalence directly is unsuitable, such level of hypertension is still too high to be paid enough attention by the government. It is also shown in former study in Chinese population that the prevalence was higher in successive age groups [18], so ageing of population may increase this rising tendency. In conclusion, our results suggest that hypertension prevalence rate is still in high level in 2014, which remains a provincial public health problem.

It was also found that hypertension prevalence of the male was higher than that of the female. Indeed, we found that the current smoking rate was significant higher in men than in women, and there were more people overweight and obese in the male. Because smoking and BMI status are found as risk factors of hypertension [19], tobacco reduction and body weight control must be firstly introduced in future policy formulated by the local government.

Former studies at the national level have discovered that hypertension among rural population increased faster than urban population from 2002 to 2010 [20]; In 2014, the hypertension prevalence in urban residents was lower than in rural residents in Jiangsu province; These all suggest that hypertension is more prevalent in rural areas. Economic improvement may be an important reason of this phenomenon, economic improvement in rural areas of China have deeply changed the living standard and economic capacity of the residents there, and risk factors grow dramatically. For instance, obesity and overweight of rural population were increasing faster than those of urban residents by improvement of economic capacity [21].

The control of blood pressure to normal level can reduce the risk of cardiovascular diseases and prevent these vascular events [22]. Our study have found that among those aware of their hypertension condition, blood pressure control rate of the whole Jiangsu residents was still low, only 23.7 % which was even lower than that of neighboring Shangdong province (43.2 %), and much lower than that found in the United States [23, 24]. The comparisons on hypertension awareness and treatment of population in different areas and with different sexual distinctions also derive an imbalance, which mainly include the rural and the male have much lower awareness and treatment level.

The control of hypertension is closely related to the factors such as, awareness of blood pressure level, the technical levels of medical staffs, patients’ medication adherence and life styles [9]. Firstly, the hypertension awareness of those residents who were classified as hypertension was rather low, so the undiscovered hypertensive patients accounted for a significant proportion, which indicate that the examination of hypertension is not still fully covered by current health service. Secondary, current self-reported anti-hypertension medication rate (88.4 %) was optimistic and acceptable, reflecting that high accessibility of treatment services have developed in Jiangsu province. At last, the hypertension control rate (23.7 %) was still low among those who underwent anti-hypertension medications, probably, treatment methods and patients’ medication adherence co-influenced the effect of therapy. In brief, both the blood pressure screening and the effect of medication should be emphasized to increase hypertension control in policies of the future National Salt Reduction Action.

In our survey, we found that the mean of salt consumption converted from urinary sodium in 24-h urine collection was 11.0 g, and only 8.6 % of all residents’ salt intake was below the recommended level (6 g) according to the dietary intake guidelines of Chinese residents [7]. The attention of salt reduction for hypertension control has been increasing throughout the world [25], and the World Health Organization (WHO) has recommended that adults consume no more than 5 g of salt per day (equivalent to 2000 mg of sodium) in 2003 [26]. In addition, total salt intake in our study would be estimated to 12.2 g when considering that approximately 10 % of sodium was excreted in sweat and feces, so excess sodium intake is prevalent in Jiangsu province.

To develop future intervention for control of hypertension, we need to combine our findings and those in other studies to adopt one advance and comprehensive method. Interestingly, the association between residents’ knowledge and attitudes of salt and their behaviors of actual salt consumption is not clear, which suggests that interventions focused only on knowledge, attitudes and behaviors may be of limited efficacy [27]. As being a traditional measure, a single intervention by a health care provider, such as the propaganda of high blood pressure knowledge, can positively impact lifestyle choices, but the extent of this impact was relatively small [28]. So a combination with salt reduction action should draw the attention of governments, and compulsory measure should be put to use. In fact, despite modest effect of dietary sodium restriction cultivated by government, the diet without addition of salt significantly decreased systolic and diastolic BP, and it should be advised for every hypertensive patient [29].

In other country, a gradual sodium reduction (10–15 % over multiple years) strategy leading to the adjustment of salt taste of the population was found valid and suitable [30]. In England, packaged food producers were the main salt using departments [31]. Differently, the major sources of sodium intake are condiments added during house cooking in China [32], so health education and health promotion should widely affect cooking habits of many housewives, such as the generation of quantitative salt spoon.

For the people unwilling or fail to taste ease, low sodium salt as an alternative is undoubtedly more attractive. A kind of salt with reduced sodium and increased potassium is now being used in northern Chinese provinces [33]. Salt substitute might be a perfect strategy to reduce sodium consumption and control hypertension. The interdependency of Na and K in the pathogenesis of hypertension indicates that Na restriction and increased K intake are important strategies for the primary prevention and treatment of hypertension and its cardiovascular consequences [32].

It was the first time to conduct large scale investigation of 24-h urinary samples in Jiangsu province. Our team has established an impeccable quality-control system for all links, which is valuable for future population studies. However, our study has also faced several limitations. Firstly, large representative sample was difficult to be involved in our completed survey. Non-responding participants were not substituted by randomly selected individuals. Only 6 counties were selected around the whole province in the first sampling stage, which also made the uncertainty bigger in estimation of 95 % CI. Secondly, the stability of electronic sphygmomanometer is not as reliable as mercurial sphygmomanometer, and blood pressure measurements on a single occasion (although 3 measurements were taken on that 1 occasion) could not substitute a clinical diagnosis of hypertension. These all increased the bias in blood pressure level measurement. Lastly, creatinine was used to substitute PABA as the biomarker for judging the completeness of 24-h urinary samples, which might have resulted in the inclusion of noneligible samples or the exclusion of eligible samples.

## Conclusion

The overall prevalence of hypertension increased significantly in Jiangsu province between 2002 and 2010, we found it was still in the high level in 2014, and the overall awareness and control of hypertension were still in unacceptably low level. While the sodium intake was seriously above the recommended level. These observations suggest that a public health approach guided by government, which including sodium intake reduction and other strategies, is urgently needed to make substantial gains in the prevention and management of hypertension-related morbidity and mortality [34].

## Ethical approval and consent

An ethical reviewing application of this program was submitted by the design team, and lately approved by the Ethical Committee of Chinese Center of Disease Control and Prevention (CDC). The number of ethical approval is 201311.

## Additional file

Additional file 1:
**PERSONAL QUESTIONNAIRE.** (DOCX 24 kb)
