# Supplementary material for: Prevalence, awareness, treatment and control of hypertension and sodium intake in Jiangsu Province, China: a baseline study in 2014
Source: BMC Public Health. 2016 Jan 21;16:56. doi: 10.1186/s12889-016-2712-y (PMC4721117; doi:10.1186/s12889-016-2712-y)
Supplement: Additional file 1: — PERSONAL QUESTIONNAIRE. (DOCX 24 kb) [file 12889_2016_2712_MOESM1_ESM.docx]

**B1. Diagnosis and control of hypertension**

Question 1: When is the last time you measure blood pressure? (before the survey)

(1) not checked

(2) within 3 months

(3) within 6 months

(4) within 12 months

(5) within more than 12 months

(99) unknown

Question 2: Do you know the level of your blood pressure?

(1) above the normal range

(2) normally

(3) below the normal range

(99) unknown

Question 3: Would you be diagnosed with high blood pressure by the doctor?

(1)yes

(2) no (jump to B2)

(99) unknown (jump to B2)

Question 4: When you diagnosed with high blood pressure?

/ / (year/month) (" unknown "fill in" 99 ")

Question 4.1: What is the level of medical institutions where you diagnosed as hypertention, (the highest level)?

(1) at the provincial hospitals and above

(2) the municipal hospital

(3) the county hospital

(4) township hospital

(5) village clinic

(88) Others

(99) unknown

Question 5: what measures do you take to control hypertention (multiple choice)?

(1) taking hypotensive drugs

(2) quitting drinking

(3) less alcohol

(4) eat less salt

(5) more exercise

(6) adjust psychological

(7) traditional Chinese medicine

(8) all not

(88)others

(99) unknown

Question 6: Did you took antihypertensive drugs in recent two weeks?
(1) yes

(2) not taking

Question 7: Have you attended the follow-up of hypertension management provided by primary health institutions?

(1) yes

(2) no (jump to B2)

(99) unknown (jump to B2)

Question 8: In the past 12 months, did the doctors in primary health institutions provided you with the following inspection or guidance? (multiple choice)

(1) to measure blood pressure, times/year

(2) guidelines, times/year

(3) the dietary guidelines

(4) physical activity guide

(5) quit smoking or less smoking

(7)drinking less alcohol

(6) all not

(99) unknown

**B2. Knowledge of salt and hypertension**

Question 1: Do you know the diagnosis criteria of hypertension?

1. 140/90 mm Hg

2. 130/80 mm Hg

3. 120/80 mm Hg

4. Don't know

Question 2: Do you know the consequences caused by hypertension [multiple choices]?

1. Stroke

2. Coronary heart disease

3. Kidney disease

4. hypertensive heart disease

5. Eye disease

6. Don't know

Question 3: Do you know the risk factors that could lead to hypertension?

1. Overweight or obese

2. Habitual excessive alcohol drink

3. Habitual high-sodium diet

4. Hypertension family history

5. Hyperglycaemia and hyperlipidemia

6. Aging

7. Mental stress

8. Don't know

Question 4: Do you think any of the following statements are correct [multiple choices]?

1. 1t would be out of strength if we intake less salt

2. Too much salt could increase blood pressure

3. Whenever we are healthy， we do not need to reduce salt intake

4. Too much salt could lead to osteoporosis

5. Don't know

Question 5: Do you know the daily salt intake recommendation in China?

1. <2 g

2. <6 g

3. <9 g

4. <12 g

5. Don't know

Question 6: Do you know that reducing salt intake could influence blood pressure?

1. elevation of pressure

2. lower blood pressure

3. make no difference

4. don’t know

Question 7: Do you know excessive salt intake could cause what disease [multiple choices]?

1. Hypertension

2. Stroke

3. Myocardial infarction

4. Kidney disease

5. Stomach cancer

6. Osteoporosis

7. Don't know

Question 8: How do you assess your salt intake?

1. Too little

2. Moderate(Please go to Question 9)

3. Too much Please go to Question 9)

Question 8.1 Do you think you will have to eat less salt?

1. yes(Please go to Question 9)
2. no
3. I don’t know(Please go to Question 9)

Question 8.2 Why aren’t you going to eat less salt [multiple choices]?
1. taste light is not good

2. eating less salt without strength

3. eat much salt harmless

4. I don't know

Question 9: Have you used a scaled salt spoon to control your salt added in cooking in your household?

1. Yes, I have

2. No, I haven't (Please go to Question 10)

3. I don't know (Please go to Question 10)

Question 9.1: Where did your family get the scaled salt spoon?

1. From the health service centers

2. It was a gift of purchasing other products

3. I bought it myself

4. Other, please clarify

Question 9.2: Do you know the method of using the scaled salt spoon?

1. Yes, I know

2. No, I don't (Please go to Question 10)

Question 9.3: Could your family use the scaled salt spoon correctly?

1. Yes, we could

2. No, we couldn't

3. Don't know

correctly use: according to the quantity of 6 grams per day per person needed to estimate the meal time with salt.

Question 10: Have you received health education on the low-sodium diet?

1. Yes, I have

2. No, I haven't (Please go to Question 11)

3. Don't know (Please go to Question 11)

Question 10.1: We will read some health education channels to you. Please select the major 3 channels that you have used to receive health education on the low-sodium diet.

1. Brochures or folding

2. Broadcast, television

3. Newspaper, magazine

4. Column

5. Health professionals

6. Family member

7. Colleagues or friends

8. Slogans

9. Other, please clarify

Question 10.2: Have you advocated the low-sodium diet to your friends or colleagues?

1. Yes, I have

2. No, I haven't

Question 11: What do you think it is necessary to low salt diet in the crowd of propaganda?
(1) it is necessary to (2) unnecessary (3) just as well

Question 12: Do you think that low-sodium diet could affect food taste?

1. Yes, it will have great effect

2. Yes, it will have moderate effect, but quite acceptable

3. No, I don't think it could affect it

4. Don't know

Question 13: Which population do you think population should adhere to the low-sodium diet (multiple choices)?

1. Patients with hypertension

2. Patients with stroke

3. Patients with coronary heart disease

4. Don't know

Question 14: What is your attitude toward the low-sodium diet?

1. Support

2. Oppose

3. Neutral

Question 15: Do you know that the food nutrition labels on food packaging (nutrients)?-
(1) know (2) don't know (Please go to Question 16)

Question 15.1: Have you noticed food labeling when you buy processed food?

1. Yes, I have

2. No, I haven't(Please go to Question 16)

Question 15.2: What is your attitude on listing the sodium content in the food label on the processed food?

1. Positive

2. Negative

3. Don't know

Question 16: Have you taken actions on salt reduction?

1. Yes, I have

2. No, I haven't (Please go to Question 17)

3. Don't know (Please go to Question 17)

Question 16.1: Which salt reduction measurements have you taken [multiple choices]?

1. Reduce added salt in cooking

□ 1 yes □2 no

2. Update salt-adding procedure in food cooking, ie, postpone salt adding once food is

ready

□ 1 yes □2 no

3. Reduce high-sodium condiments use

□ 1 yes □2 no

4. Reduce the consumption of pickles (high-sodium food)

□ 1 yes □2 no

5. Choose low-sodium processed food

□ 1 yes □2 no

6. Replace the salt with condiments that were low sodium， ie， vinegar

□ 1 yes □2 no

7. Use onion, ginger, garlic to increase the taste and reduce the salt adding

□ 1 yes □2 no

8. Other, please clarify

□ 1 yes □2 no

Question 17: Have you heard of low-sodium salt (salt substitute)?

1. Yes， 1 have

2. No， 1 haven't (End)

Question 17.1: Have you used low-sodium salt?

1. Never

2) basic need not

3) occasionally to eat
4) regular consumption (end)

5) has been eating (end)

"Never" means never eat low-sodium salt; In "basic need not" refers to eat salt is below 30% for the low sodium salt; "Occasionally eating" means eating salt of 30% ~ 59% for the low sodium salt ;"Regular consumption" means eating more than 60% of salt and for the low sodium salt; "Have been eating" means eating salt of 90% and above for the low sodium salt

Question 17.2: Could you please tell us the reason why you don't plan or less to reduce salt?

1.Price is high

2. Difficult to buy

3. the light taste, not used to

4. just as well

5. other
